# Handling Complexity in Animal and Plant Science Research—From Single to Functional Traits: Are We There Yet?

**DOI:** 10.3390/ht7020016

**Published:** 2018-05-28

**Authors:** Jessica Roberts, Aoife Power, Shaneel Chandra, James Chapman, Daniel Cozzolino

**Affiliations:** The Agri-Chemistry Group, School of Medical and Applied Sciences, Central Queensland University (CQU), Bruce Highway, North Rockhampton 4701, Australia; j.j.roberts@cqu.edu.au (J.R.); a.power@cqu.edu.au (A.P.); s.chandra@cqu.edu.au (S.C.); j.chapman@cqu.edu.au (J.C.)

**Keywords:** complexity, omics, quality, yield, crops, livestock

## Abstract

The current knowledge of the main factors governing livestock, crop and plant quality as well as yield in different species is incomplete. For example, this can be evidenced by the persistence of benchmark crop varieties for many decades in spite of the gains achieved over the same period. In recent years, it has been demonstrated that molecular breeding based on DNA markers has led to advances in breeding (animal and crops). However, these advances are not in the way that it was anticipated initially by the researcher in the field. According to several scientists, one of the main reasons for this was related to the evidence that complex target traits such as grain yield, composition or nutritional quality depend on multiple factors in addition to genetics. Therefore, some questions need to be asked: are the current approaches in molecular genetics the most appropriate to deal with complex traits such as yield or quality? Are the current tools for phenotyping complex traits enough to differentiate among genotypes? Do we need to change the way that data is collected and analysed?

## 1. Introduction

The current knowledge of the main factors regulating and controlling quality and yield in livestock, crops and plants of economic importance is incomplete. For example, this is reflected by the persistence of benchmarking crop varieties for many decades in spite of the gains achieved over the same period of time [1]. For many years, information and data provided by the current phenotyping methods have enabled breeders to select a few simple quality traits. However, new crop varieties with similar or the same breeding/phenotyping score cannot be differentiated by the consumers (e.g., rice or wheat quality) or by their different performance during processing (e.g., malt barley, carcass and meat quality). In the same way, properties such as yield can be affected by maturity, differences in vegetative phases, photoperiod, biomass accumulation, but not a single measurement can be associated or used to explain gains associated with yield [2].

It is well established that molecular breeding based on DNA markers has led to advances in breeding (crops and livestock); however, not in the way that it was anticipated initially [3]. One of the main reasons for this was that complex target traits such as grain yield, composition or nutritional quality depend on multiple factors in addition to genetics [4]. Therefore, some questions need to be asked to expand our knowledge about crop and plant genetics. Are the current approaches in molecular genetics the most appropriate to deal with complex traits such as yield and quality? Are the current tools for phenotyping complex traits enough to differentiate among genotypes? Do we need to change the way that data is collected and analysed?

The current knowledge of the main factors governing these important production traits in different genetic backgrounds is incomplete, and this is reflected by the lack of robust phenotypic information [5]. For many years, data provided by current phenotyping methods have enabled breeders, scientists and producers to select a few basic traits associated with nutrition or quality and yield [3]. Over the last century, agricultural and animal research has dramatically enhanced the production of technologies and methodologies to support the beef, dairy, swine, poultry aquaculture and sheep industries [6]. In the last 20 years, molecular biology has changed the way research is conducted in agricultural, animal and plant research, supported by the innovations and progresses in genomics and the relatively new offshoot disciplines of functional genomics, proteomics, transcriptomics, metabolomics and metagenomics [7]. Additionally, improved innovation and supplementary technologies have led to the development of physical genome maps and the publication of genetic libraries, which in turn have advanced the implicit understanding of genetics at the molecular level, particularly the different genetic components and the phenotypic variations associated with these differences [8]. Quantitative geneticists have been able to improve production traits; however, genomic technology has the potential to create accurate and rapid animal improvement based upon phenotypic traits typically difficult to measure [9,10].

Recently, different disciplines in the agricultural field have been able to exploit pioneering research built off the human-genome project by sequencing two of the major livestock genomes (*Gallus domesticus* and *Bos taurus*) [9,10,11]. This research has brought about new challenges in the agriculture disciplines associated with animal and plant breeding [9,10]. A number of improvements in efficiency of production have not come about without some serious adverse effects. These adverse effects include side-effects on well-being and longevity in the production environment, losses in reproductive efficiency, increased levels of stress, increased animal wastage and waste issues, as well as increasing rates of infectious diseases [11].

When production improvement occurs, these improvements can be also related to societal concerns in areas such as natural resource conservation and protection, animal welfare and food safety [11]. It is evident that public support for agricultural research must be focused on enhancing the functionality and wellbeing of livestock and poultry in environmentally neutral production systems for the future [11]. With the expected and rapid increases in knowledge in agriculture research, it is imperative that methodologies for defining phenotypes are clear and standardized [11]. Furthermore, the detection of any mutations or altered expressions of genes depends on phenotypic screening methods and the ability to detect variations from normal [12]. The next challenge will be to develop fast, efficient, systematic and comprehensive phenotypic screening procedures and tools that will permit comparison among laboratories [12]. It is well established that molecular breeding based on DNA markers has led to advances in breeding; however, not in the way that it was anticipated initially [13]. One of the main reasons for this was that complex target traits such as feed intake, behavior, disease resistance, composition or nutritional quality depend on multiple factors in addition to genetics [14]. 

In recent years, most of the scientific literature (research and review papers) concentrates on the methods or benefits of the omics approach (e.g., proteomics, lipidomics, metabolomics) [15]. However, the interpretation of the resultant data remains reductionist. Animals/livestock are the sources of an almost uncountable number of metabolites whose structure, function and usability have been explored only partially [15]. Recently, the concept of one-gene, one-mRNA, one-protein analysis has been reduced as methodologies endeavor to measure more complex traits [16,17]. 

The life science disciplines are addressing a number of complex global biological systems and systems which are related to the omic technologies. These omic technologies have fast become a growing area of interest for many researchers [18]. For example, metabolomics are considered to be the functional part of the omic sciences, being heralded as an effective tool for assessing biochemical processes relating to complex [19]. In contrast to transcriptome or proteome, the metabolome is chemically and physically more diverse owing to large variations in atomic arrangements [15].

High throughput and robust analytical techniques are implemented to assess the global metabolic profile. The use of several analytical techniques based on spectroscopy, mass spectrometry (MS), liquid chromatography (LC), inductively coupled plasma (ICP) [20], vibrational spectroscopy methods [21] (e.g., mid-infrared, near infrared) [22], as well as hyphenated techniques has undoubtedly strengthened the field of omics, allowing a more holistic analysis of the different biological systems [23]. However, researchers still use the reductionist approach, looking for individual or group of markers/properties/traits and not using the advantages provided by these holistic techniques. An essential characteristic of metabolomics research is that the sample preparation remains a critical issue because, if inconsistent techniques are used, these techniques will generate unreliable results and large sources of error. It is important to realise that a large depth of understanding of the physiology of single plant species for practical applications as well as translating this acquired knowledge into complex natural as well as anthropogenic ecosystems is required when dealing with metabolomics research [23].

Bioanalytical and biological research developments will lead to paradigm-changing understanding in trying to appreciate organisms as a level outside of their ecosystem context. Importantly, the shotgun and next generation genome sequencing, gene reconstruction and gene annotation as well as genome-scale molecular analysis using some of the omic technologies described will produce computer-assisted analysis and modelling for biological data [24].

In systems biology, molecular data, genetic evolution, environmental cues and species interactions, modelling and prediction of active biochemical networks with whole species populations are combined [24,25,26,27,28]. This combinatory process relies on the development of new technologies and methods for the analysis of molecular data, especially genomics, metabolomics and proteomics data [24,25,26,27,28]. By integrating genotyping, pheno/morphotyping and the analysis of the molecular phenotype using metabolomics, proteomics and transcriptomics, this will reveal a unique understanding of plant metabolism and its interaction with the environment. In the analysis of single model systems—plants, fungi, animals and bacteria—a model will finally emerge in the analysis of populations of plants and other organisms and their adaptation to the ecological niche [24,25,26,27,28]. This understanding of ecophysiology will translate into knowledge-based approaches in crop plant biotechnology and marker- or genome-assisted breeding approaches [25]. Therefore, a metabolomic study, producing information-rich, highly reliable and reproducible data-sets in non-targeted or global and multivariate statistical analysis can be achieved. Metabolomics therefore represents, in a new way, the ability to dissect and modify plant metabolism, physiology and development [29]. These capabilities will be essential in breeding more robust plant varieties [29]. Functional genomics, as the name implies, aims to decipher gene function by establishing a better undertaking of the correlations between genes and the functional phenotype [29]. Functional genomics will produce smarter genomics rather than simply gene mapping and sequencing, and the motivation for this research endeavour arises because of the proportion of open reading frames in a fully sequenced organism that have no known function at the biochemical and phenotype level [29]. Table 1 shows the applications of metabolomics where a different approach in terms of the experimental design, sampling protocol and data analysis will be required in order to further progress in our understanding of biological systems.

## 2. Omics, Metabolomics and Systems Biology

Most of the scientific literature and reviews on the topic concentrate on the methods or benefits of the omics approach. However, researchers still use the reductionist approach, looking for individual or groups of markers, and not using the advantages provided by these holistic techniques [26,27]. For example, these methods have been developed and implemented as an important tool for monitoring and quantifying the number of metabolites induced by the interactions between genotype, terroir, viticultural/management practices and the winemaking processes in grapes [26,27]. Moreover, some of the current trends of metabolomics focus on the study of alterations in metabolic pathways stemming from grapevine diseases or genetically modified cultivars, as well as management practices in the vineyard [28].

An important characteristic of metabolomics is that sample preparation is an important issue, mainly because if inconsistent techniques are used they will generate unreliable results and invalid sources of error [28]. Recently, systems biology has undergone a clear shift to focussing on crop and plant abiotic stress [30]. While crops and plants are exposed to a myriad of abiotic stresses throughout their lifetimes, a tangible understanding of the abiotic stress responses and tolerance of economically valuable crop species are essential for their domestication and to maximize their yield in future climate scenarios [30]. While crop domestication and abiotic stress research is by no means a recent phenomenon, recent developments in next-generation genome sequencing platforms and functional genomics studies have caught the imagination of researchers in the field [30]. Unsurprisingly, this has resulted in multiple research teams exploiting such platforms to better understand the complex dynamics of abiotic stress tolerance in plants. These groups to date have emphasized the role of a number of genetic markers including random amplification of polymorphic DNA (RAPD), restriction fragment length polymorphism (RFLP), amplified fragment length polymorphism (AFLP), simple sequence repeat (SSR), and single nucleotide polymorphisms (SNPs) and have reported the potential of such research to facilitate the advancement of genomic research, which in turn has led to the discovery of novel agronomic traits [30]. Moreover, the incorporation of Bayesian models has allowed the simultaneous discovery of multiple molecular markers, which in turn has provided precise information about novel quantitative trait loci (QTLs) and epistasis [30]. Thus, in the authors’ opinion, it is evident that the use of a systems approach has enabled the establishment of comprehensive molecular links between phenotype and genetic variations [30]. Such studies have further benefited the discipline via the development of comprehensive datasets and libraries for plant genotype and phenotypic variants, valuable tools to aid the understanding of abiotic stress related plant phenotypes [30]. In addition, the coupling of genomics with metabolomics contributes to the potential of metabolic engineering of favorable agronomic traits. As such, it is not unrealistic to expect that the integration of multiple omics technologies with cutting edge co-expression interaction analysis of genes will accelerate abiotic stress tolerance research in the short term [30].

Recently, fluxomes have been incorporated as further lenses to “omics analyses” [31,32,33,34,35,36]. This addition is important when considering the dynamic relationships between proteome and metabolome in plant metabolomics [31,32,33,34,35,36]. Their interaction can be exploited to determine the rates of growth and product formation by monitoring the steady state rates of significant cellular phenotypes during their metabolic inter conversion within living cells [29]. Such analysis requires the determination of the steady-state flux distribution, which in turn is calculated using flux balance analysis: this approach relies on the quantification of a set of experimentally measured metabolic fluxes within a network, such as production excretion or substrate consumption [31,32,33,34,35,36]. This fluxome process may also be referred to as metabolic regulon, in which the system’s innate control of metabolite levels through the regulation of metabolic flux of the biosynthesis and catabolism pathway is impaired [31,32,33,34,35,36]. Consequently, a deeper understanding of the modes of regulation within a plant’s metabolic system can be achieved through a quantitative investigation of its metabolic flux [31,37]. This can be measured using enzyme assay platforms which allow the quantitative estimation of gene expression levels. [31,38]. Moreover, the use of enzyme assay platforms more directly elucidate the metabolic pathway than transcriptome data [31] (the traditional approach), as it allows the measurement of the “net” activity of each (including multiple isoenzymes) reaction step. Recent studies have also highlighted that the utilization of dynamic labeling with the 13C isotope is to be a powerful tool for the elucidation of the metabolic regulon mechanism [31]. The incorporation of such has resulted in the investigation of various dynamics aspects of plant metabolism [31].

## 3. From Reductionist to Omics Approach

Nowadays, in crop and plant science, a new paradigm shift has become a reality: the use of high throughput methods allowing the collection of hundreds of data points which can be related to many applications. However, this approach is still focused on measuring single and simple traits where the focus has been on the reductionist approach. This approach has prompted an “unreal worldview” where single factors have been analyzed independently of the matrix as a whole [39]. Consequently, this reductionist approach is no longer deemed useful in many research fields [39]. For example, in cereal science, a reductionist approach has led breeders to select varieties based on only one or few characteristics (e.g., high amylose, protein, bet glucan) [39]. However, more researchers are considering the hypothesis that the “whole grain” (fingerprint) as a package needs to be considered instead of targeting specific components. The thought process is based on the fact that complex systems require complex answers, and many research fields must move towards a more holistic and integrative approach in order to generate new and novel knowledge [39].

Invariably, the best crop production processes are those grounded in scientific research, as they are, by nature, continually evolving and improving [40]. This success will depend upon the establishment of strategic alliances between the plant physiologist, the biochemical researchers, the biostatisticians, the breeders and the agronomist [41]. Therefore, this holistic approach towards using omics serves as an invaluable tool in modern plant analysis as the omics methods acquire the broadest overview of a sample’s metabolic composition [42]. Every metabolic study should establish an unbiased, integrated strategy that addresses the number of issues of sample preparation, data treatment, metabolite identification and quantification [43,44]. 

## 4. Limitations

Many relationships in data cannot be expressed in quantitative terms, and these links are better expressed concerning similarity or dissimilarity and among a group of samples (patterns) [45]. A recent study has highlighted the fact that changes in the metabolite profile of corn (*Zea mays*) are more closely related to the agronomic phenotype than any chosen fragment of nucleic acid [46]. Therefore, there is a need to enhance our knowledge about the relevant components that can affect quality and yield traits and also develop new tools to enable the improvement of those characteristics [47]. These studies are normally related to multifactorial issues, and it is in this context that it makes good sense to explore and to measure the same sample on complementary, synergistic and non-destructive analytical platforms that comprise multifactorial sensors and separation methods. The difficulty of exploring, extracting and describing the data in this way increases the challenge exponentially as well as increasing the risk of becoming flooded with non-informative data increases concomitantly.

The acquisition of data from different analytical platforms provides researchers with new opportunities in food research. The issues such as the validity of the information from the data generated, the comparison of the data between different analytical platforms and the need for rigorous control of the integrity of the data in the context of the models generated are still the primary constraints facing “omic” approaches in the omic revolution [45]. The limitations created by the current state of the art techniques in bioinformatic tools, the limited information in food databases (e.g., on the identity of many metabolites), our still poor knowledge on many molecular processes taking place in cells, and also the difficulty to combine huge data generated by the so called “omics” technologies such as transcriptomics, proteomics and metabolomics (e.g., systems biology) are still critical to this discussion [48]. As reported by other authors, the need for long term investigation is essential in order to achieve the necessary perspective (and knowledge) on these complex and fundamental topics [39,40,41,42,43,44,45,46,47,48,49].

For example, both yield and quality are not single traits; they are complex combinations of individual traits. Therefore, the efforts in understanding these complex characteristics with the current tools raise the following question: is the reductionist approach the most adequate? Do we need to change our approach?

Models can be constructed from different perspectives as outlined above, and for each type of model, several approaches are available [50,51,52,53,54,55]. No single approach will explain the complex interactions in biological networks entirely. Often, a number of techniques are used. When attempting to capture plant performance over multiple levels of complexity, models are needed that can cope with different regulatory mechanisms. In top-down models, statistical modelling is often the go-to technique, whereas in bottom–up models, dynamic or constraint-based modelling seems to be appropriate [50,51,52,53,54,55]. However, these tools are not restricted to a particular class of model and are used synergistically and frequently: e.g., multivariate statistics and machine learning techniques might be used to reconstruct a network topology in the first step of the modelling cycle, whereas dynamic models might be used to predict the dynamic behavior of the network in subsequent steps. Subsequently, at the genome-scale, constraint-based models of metabolism can be built bottom–up from genomic, biochemical and thermodynamic information [49].

The statistical network models described can be derived via regression techniques, relevance systems based on association scores, Gaussian graphical models allowing identification of conditional independence, or Bayesian networks used to represent probabilistic relationships [49]. The complexity of biological networks makes comprehensive experimental testing not always feasible, but computational models can be helpful in predicting the outcomes of different scenarios and thereby assist experimental design by pinpointing the most promising strategies and reducing the distance in the end possibilities [51]. Modern research will significantly benefit from intense cooperation between experimentalists and modelers. Moreover, such models can be utilized to predict which parameters to alter to achieve the desired system performance, a premise highly demanded by the agricultural community. Such an interdisciplinary approach will deepen our understanding of the functioning of plants as individuals in complex environments, including the underlying mechanisms, and the consequences of their phenotypes for the functioning of plants in the context of interactions with other organisms in complex biological communities [52].

## 5. Conclusions

In recent decades, significant advances in instrumentation (hardware) and multivariate data manipulation techniques (e.g., new algorithms, software) have allowed the development of novel “omic” applications [52,53,54,55]. Despite multiple publications in the scientific literature regarding these “omic” developments, there remains a clear gap between the robust real-world application of such high throughput technologies and feasibility studies. This is a consequence of various roadblocks that still hinder the growth and uptake of these applications, such as the hesitancy of industry to accept the integration of chemistry and mathematics (the benefits of chemometrics are often ignored by those who prefer to employ classical statistics), the lack of formal (academic) education in the use and application of instrumental methods as high throughput tools or on the implementation of an holistic approach to complex systems analysis (the traditional reductionist approach is generally favored). The authors have little doubt that biggest challenge stifling the implementation and development of omic implementations is not the ability to interpret the information derived from high throughput tools, and the mathematical models generated through multivariate analysis, but the skepticism and reluctance of a large portion of the research community to do so. 

## Figures and Tables

**Table 1:** The application of metabolomics will require a different approach in terms of the experimental design, sampling protocol and data analysis.

Main Topic	Issues to be Addressed
Field trials	Robust field trials
Sampling protocol	Define a correct protocol, replicates, consistency
Validation	Define a validation protocol transfer models, information, internal and external validation
Pre-processing	Consistency, interpretation and validation
Appropriate method bioinformatics/chemometrics	Consistency, interpretation and validation
Analytical method	Error, repeatability and validation
Education	Graduates and users
Multidisciplinary approach	Integration of different disciplines
